# *Saccharomyces boulardii *ingestion increases the humoral response of a DNA vaccine against leptospirosis in mice

**DOI:** 10.1186/1753-6561-8-S4-P160

**Published:** 2014-10-01

**Authors:** Marcelle Silveira, Rafael Rosa, Marcelo Mendonça, Daiane Hartwig, Alexandre Bilhalva, Gustavo Moreira, Patrícia Diaz, Luisa Scapin, Fabrício Conceição, Ângela Moreira

**Affiliations:** 1 Centro de Desenvolvimento Tecnológico, Núcleo de Biotecnologia, Universidade Federal de Pelotas, Pelotas, RS, Brazil; 2 Instituto de Biologia, Departamento de microbiologia e parasitologia, Universidade Federal de Pelotas, Pelotas, RS, Brazil; 3 Engenharia de materiais, CDTec, Universidade Federal de Pelotas, Pelotas, RS, Brazil; 4 Faculdade de Nutrição, Universidade Federal de Pelotas, Pelotas, RS, Brazil

## Background

DNA vaccines are a good option to generate a desired antigen using the cellular machinery of the vaccinated subject. This kind of vaccine can induce both of the humoral and cellular immune response, and show high stability and easy working, as well as offer a low-cost production. and safety for immune compromised patients [1]. However, some disadvantages, such as the low transfection rate and low immunogenicity, makes necessary the use of multiple doses [2,3]. Therefore, several studies have been conducted trying to increase the immune response generated by the DNA vaccines. *Sacharomyces boulardii*, a probiotic yeast that is capable to increase the host immune response [4], was not previously evaluated as an adjuvant for DNA vaccines. Thereby, in the present study we evaluated the *S. boulardii *capacity to increase the humoral response of mice using DNA vaccines against a fragment of the leptospiral antigen LigAcloned in the mammalian expression plasmid pTARGET [4,5].

## Material and methods

Four to six month-old Female BALB/c mice were separated into 2 groups of 12 animals each. Group 1 (G1, control) were fed with antimicrobial-free ration; group 2 (G2) with the same ration containing 10^8^cfu.g^-1 ^of *S. boulardii*, 14 days before the first immunization, for adaptation, and during all the experiment. The immunization protocol consisted in 3 doses (days 0, 14 and 21) of 100 µg of the pTARGET/*lig*Ani plasmid injected intramuscularly. Blood samples were collected from the retro eye venous plexus of the animals on days 0, 13, 20 and 27 for the humoral immune response evaluation of each immunization through indirect ELISA, using recombinant LigAni (5 µg.mL^-1^) as antigen and serum dilutions 1:30 in PBS-T.ELISA units were calculated by dividing the mean absorbance of each group by the mean absorbance of the day 0. The student's test-t was performed to determine significant differences (p < 0.05) between the groups.

## Results and conclusions

Sera from G2 has shown ELISA units statistically higher than the sera from G1 after the second (p < 0.0001) and third immunization (p = 0.0264). In addition, there was nosignificant difference between the second and third immunizations of G2. This way, the results indicate that using *S. boulardii *as adjuvant, only two immunizations are sufficient to obtain an adequate immune response. Therefore, the *S. boulardii *probiotic was able to enhance the humoral immune response to a DNA vaccine against leptospirosis in mice.
